# Persistence of sewage-associated genetic markers in advanced and conventional treated recycled water: implications for microbial source tracking in surface waters

**DOI:** 10.1128/mbio.00655-24

**Published:** 2024-06-12

**Authors:** Aldo E. Lobos, Amanda M. Brandt, Javier F. Gallard-Góngora, Ruchi Korde, Eleanor Brodrick, Valerie J. Harwood

**Affiliations:** 1 Department of Integrative Biology, University of South Florida, Tampa, Florida, USA; 2 Department of Earth, Marine, and Environmental Sciences, Institute of Marine Science, University of North Carolina at Chapel Hill, Morehead City, North Carolina, USA; University of Tennessee at Knoxville, Knoxville, Tennessee, USA

**Keywords:** reclaimed water, water quality, recreational water, health risk, fecal indicator, anthropogenic pollution, WWTP

## Abstract

Sewage contamination of environmental waters is increasingly assessed by measuring DNA from sewage-associated microorganisms in microbial source tracking (MST) approaches. However, DNA can persist through wastewater treatment and reach surface waters when treated sewage/recycled water is discharged, which may falsely indicate pollution from untreated sewage. Recycled water discharged from an advanced wastewater treatment (AWT) facility into a Florida stream elevated the sewage-associated HF183 marker 1,000-fold, with a minimal increase in cultured *Escherichia coli*. The persistence of sewage-associated microorganisms was compared by qPCR in untreated sewage and recycled water from conventional wastewater treatment (CWT) and AWT facilities. *E. coli* (EC23S857) and sewage-associated markers HF183, H8, and viral crAssphage CPQ_056 were always detected in untreated sewage (6.5–8.7 log_10_ GC/100 mL). Multivariate analysis found a significantly greater reduction of microbial variables via AWT vs CWT. Bacterial markers decayed ~4–5 log_10_ through CWT, but CPQ_056 was ~100-fold more persistent. In AWT facilities, the log_10_ reduction of all variables was ~5. In recycled water, bacterial marker concentrations were significantly correlated (*P* ≤ 0.0136; tau ≥ 0.44); however, CPQ_056 was not correlated with any marker, suggesting varying drivers of decay. Concentrations of cultured *E. coli* carrying the H8 marker (EcH8) in untreated sewage were 5.24–6.02 log_10_ CFU/100 mL, while no *E. coli* was isolated from recycled water. HF183 and culturable EcH8 were also correlated in contaminated surface waters (odds ratio *β*_1_ = 1.701). Culturable EcH8 has a strong potential to differentiate positive MST marker signals arising from treated (e.g., recycled water) and untreated sewage discharged into environmental waters.

**IMPORTANCE:**

Genes in sewage-associated microorganisms are widely accepted indicators of sewage pollution in environmental waters. However, DNA persists through wastewater treatment and can reach surface waters when recycled water is discharged, potentially causing false-positive indications of sewage contamination. Previous studies have found that bacterial and viral sewage-associated genes persist through wastewater treatment; however, these studies did not compare different facilities or identify a solution to distinguish sewage from recycled water. In this study, we demonstrated the persistence of bacterial marker genes and the greater persistence of a viral marker gene (CPQ_056 of crAssphage) through varying wastewater treatment facilities. We also aim to provide a tool to confirm sewage contamination in surface waters with recycled water inputs. This work showed that the level of wastewater treatment affects the removal of microorganisms, particularly viruses, and expands our ability to identify sewage in surface waters.

## INTRODUCTION

Untreated sewage pollution can introduce waterborne pathogens, which are a major health concern for recreational swimmers, into surface waters (1–3). Discrimination of fecal sources has become a priority since human fecal pollution (i.e., untreated sewage) is considered a higher risk to human health compared to other fecal sources due to the presence of a high diversity of pathogens, including human-specific viruses (4, 5). If the source of pollution is not identified, remediation efforts and attempts to mitigate further pollution will likely fail. To combat the deficiencies of conventional fecal indicator bacteria such as *Escherichia coli,* which cannot discriminate among fecal sources and provide insufficient information about human health risks, microbial source tracking (MST) methods based on host-associated genetic markers have been developed (6). For example, the bacterial HF183 MST marker is strongly associated with the human gastrointestinal tract and is ubiquitous in untreated sewage in the United States (7). Measuring microbial variables such as HF183 by qPCR can also help estimate human health risk by measuring the extent of human fecal/sewage pollution and distinguishing contamination from human feces and untreated sewage from domestic or wildlife animal sources (7). One limitation of this approach is that qPCR methods lack the ability to discriminate between viable and nonviable cells, as well as free DNA, since they simply detect nucleic acid.

Differentiating recycled water from untreated sewage in surface waters is necessary for accurate risk assessment since epidemiological studies have shown a lower health risk from exposure to recycled water (8–12), while exposure to untreated sewage is a definite health risk for recreational water users (13, 14). The risk associated with exposure to recycled water by direct potable reuse is highly dependent on the treatment process utilized and the density of pathogens present in sewage prior to treatment (15). Approximately 12–15 log reduction of viruses is recommended for safe direct potable reuse of treated wastewater (i.e., recycled water); however, there is a need for more accurate methods that measure viral infectivity for risk assessments (16). In recreational waters, the reduction of viruses in recycled water required for safe conditions may be lower (16); however, the required level of treatment is unclear. Despite this knowledge gap, it remains imperative that we can distinguish untreated sewage from recycled water in the environment to prioritize sources of fecal microorganisms in environmental waters and better inform future epidemiology studies and risk assessments.

Recycled water containing quantifiable levels of sewage-associated MST markers may interfere with MST efforts in environmental waters focused on the identification of only untreated sewage. In addition to discharge into surface waters, recycled water can be redirected and used for irrigation, groundwater recharge, or other applications (17). Recycled water applications with a strong potential to impact surface water, such as lawn irrigation, are prevalent in many states in the United States, including Florida. An estimated minimum of 900 million gallons per day of recycled water is utilized by Florida alone in various land applications, such as edible crops (6,000 acres), 500 golf courses, 1,000 schools, and 500,000 residences (18). Recycled water that enters surface waters can contribute DNA from compromised or dead cells to the environment (19, 20). Previous studies have found that two sewage-associated viral genetic markers (crAssphage and pepper mild mottle virus), the bacterial marker HF183, and antibiotic resistance genes persist through the production of recycled water (19, 21–23).

Recycled water can be produced in advanced or conventional wastewater treatment facilities (WWTFs), which vary in the level of treatment. Conventional wastewater treatment (CWT) facilities in the United States typically employ primary (physical) and secondary (biological) treatment, followed by disinfection. Advanced wastewater treatment (AWT) can include multiple approaches to reduce the levels of nutrients and microbial contaminants in secondary-treated sewage effluent, e.g., adsorption, dual media filters, denitrification filters, membrane filtration, membrane bioreactor, flocculation, biological aerated filter, chemical oxidation, and Bardenpho processes (24, 25). Persistence through wastewater treatment is generally reported by measuring the frequency of detection or decay (e.g., log_10_ reduction) of microbial analytes. Previous studies demonstrated that AWT can further reduce concentrations of pathogens (e.g., *Cryptosporidium*, *E. coli*, enterovirus, *Giardia*, norovirus, and *Salmonella*) and antibiotic-resistant bacteria (24, 26–29) compared to CWT. However, the literature lacks evidence for the relative efficacy of the removal of sewage-associated genetic markers in AWT vs CWT facilities. A deeper understanding of these differences is necessary to guide MST efforts and to inform decisions on the treatment process used to produce the recycled water that ultimately enters environmental surface waters.

Improving methods to quantify infectious pathogens in surface waters is an ongoing effort that is crucial to accurate health risk assessments, which frequently rely on measuring surrogates by qPCR. No widely accepted method to eliminate DNA, and thus the qPCR signal, from dead cells in treated wastewater has been established. Some studies have utilized techniques (e.g., propidium monoazide treatment, PMA) that attempt to prevent the amplification of DNA from non-viable cells in qPCR tests (30, 31); however, constituents such as total suspended solids can interfere with light activation of the PMA dye, and a major knowledge gap remains on the dye’s ability to penetrate cell membranes of viable but non-culturable cells (32). Even if these techniques could fully attenuate PCR amplification in viable but non-culturable cells, they lack the ability to eliminate a qPCR signal originating from dead cells or free DNA (extracellular) (33), further confounding the live/dead interpretation. A method that combines cultivation and genetic techniques to quantify sewage-associated bacterial genetic markers may provide a solution for the discrimination of untreated sewage from recycled water in treated waste flows and environmental surface waters.

Viable *E. coli* is consistently present in untreated sewage and is generally not culturable in recycled water. Four gene fragments in *E. coli* strains associated with human feces were previously identified by whole-genome sequencing (34). A performance study of culturable *E. coli* containing these genes was conducted in the United States and found that the H8 gene, a sodium/hydrogen exchanger precursor, had the highest sensitivity (percentage of target, i.e., sewage and human fecal samples, positive for the targeted gene) and specificity [percentage of non-target (fecal samples from animals other than human) samples negative for sewage-associated gene] among the four genes tested for tracking untreated sewage pollution in sub-tropical surface waters (35). These methods may have utility to identify viable *E. coli* cells originating from untreated sewage without confounding target DNA from extracellular and dead cells in recycled water by including an enrichment step to test *E. coli* isolates for the H8 marker.

We tested three major hypotheses in this study: the first two aimed at exploring the potential for recycled water to produce positive indications of untreated sewage pollution in environmental waters tested by qPCR methods, and the third tested an alternative method based on culture of *E. coli* followed by probe-based real-time PCR to detect the H8 gene (culturable EcH8). The hypotheses addressed are (i) recycled water contains levels of sewage-associated MST marker genes that could influence MST analysis of fecal sources in surface waters, (ii) persistence of sewage-associated MST genes in recycled water is reduced in AWT compared to CWT facilities, and (iii) culturable *E. coli* containing the H8 gene is consistently present in untreated sewage and absent in recycled water. To test these hypotheses, we examined the differences in MST marker gene concentrations and compared their persistence by frequency of detection and log_10_ reduction in untreated sewage and recycled water from three AWT and three CWT facilities in Florida, USA.

## MATERIALS AND METHODS

### Sites and sampling

Three types of experiments were carried out in this study. We examined (i) the effect of recycled water discharge on levels of HF183 and culturable *E. coli*, (ii) the persistence of MST markers through AWT and CWT, and (iii) conducted a surface water survey to assess the relationship between HF183 and culturable EcH8. In experiment i, the effect of the input of recycled water discharged from an AWT facility on culturable *E. coli* and HF183 levels in Turkey Creek, a first-order Florida stream, was evaluated during a discharge event. Samples were collected 7 days before, during, and 23 hours after a scheduled recycled water discharge from three sites: the discharge point (latitude: 27.955679, longitude: −82.20926), from which water flows along a canal into the creek, a site 1.21 km downstream, which was impacted by the discharged effluent, and a site 0.24 km upstream of the confluence and is not affected by the discharge (Fig. S1). Water samples were collected in sterilized, 1 L polypropylene bottles, transported on ice, and processed within 2 hours.

In experiment ii, untreated sewage and recycled water were collected between March and September 2021 from three AWT (facilities D–F) and CWT (facilities A–C) facilities in Tampa and St. Petersburg, FL, USA (Table 1). Each WWTF location was sampled three times, yielding 18 untreated sewage samples and 18 recycled water (treated) samples. Untreated sewage (500 mL) and recycled water (2 L) samples were collected in sterile polypropylene containers and transported at 4°C on wet ice to the laboratory. Samples were processed within 6 hours for analysis of culturable *E. coli* and DNA extraction.

**TABLE 1:** Characteristics of advanced and conventional wastewater treatment facilities^*a*^

Facility	WWTF capacity (MGD)	Recycled water produced (MGD)	Estimated population served	Treatment processes	Disinfection
Conventional					
A	20	16.4	126,880	Primary/secondary	Sodium hypochlorite
				Activated sludge treatment	
B	20	10	80,560	Primary/secondary	Sodium hypochlorite
				Activated sludge treatment	
C	16	8	62,320	Primary/secondary	Sodium hypochlorite
				Activated sludge treatment	
Advanced					
D	12	9.1	172,346	(i) Primary/secondary	UV
				(ii) Anoxic basins and oxidation ditches	
				(iii) Dual media deep-bed denitrification filters	
E	33	14.6	200,000	(i) Primary/secondary	Chlorine and sodium hypochlorite
				(ii) Activated sludge treatment in an anoxic/aerobic configuration	
				(iii) Deep-bed denitrification filters with methanol addition	
F	9	6.5	100,000	(i) Primary/secondary	Chlorine
				(ii) Five-stage Bardenphoactivated sludge process	

^*a*^ All untreated sewage influent samples were collected before treatment, while all recycled water samples were collected post-disinfection and prior to entering the distribution system.

In experiment iii, surface water samples from eight water bodies in St. Petersburg, FL, USA were collected monthly for 2 years. These sites are classified as impaired waterbodies due to the consistent exceedance of recreational water quality criteria for fecal indicator bacteria levels, which may have been influenced by untreated sewage inputs. The area was also served by irrigation lines delivering recycled water. Samples (500 mL) were stored for up to 2 hours in sterile 500 mL Nalgene containers on wet ice and processed by membrane filtration for the cultivation of *E. coli* and environmental DNA extraction.

### *E. coli* culture

Surface water samples were concentrated in duplicate using three volumes (0.1, 1, and 10 mL) onto mixed cellulose ester filters (47 mm diameter, 0.45 µm pore size; Fisherbrand) by membrane filtration. *E. coli* was cultured from the samples and enumerated on mTEC utilizing USEPA Method 1603 (36). In addition, a phosphate-buffered saline (PBS, pH 7.0) blank was filtered and plated on mTEC to check for contamination. Prior to each sampling event, mTEC agar was tested with a positive control (*E. coli*, ATCC 11775) and a negative control (*Enterococcus faecalis*, ATCC 19433). *E. coli* concentrations were reported in CFU/100 mL. The limit of detection for culturable *E. coli* in surface waters was 1 × 10^1^ CFU/100 mL.

Bacteria from untreated sewage and recycled water were concentrated by membrane filtration as described above. Untreated sewage samples were diluted 10^−3^-fold, and 1 mL was filtered (equivalent to 0.001 mL of untreated sewage). One liter of each recycled water sample was concentrated by filtration. *E. coli* was cultured and enumerated as described above. The limit of detection for culturable *E. coli* in this study was 1.0 × 10^5^ CFU/100 mL for untreated sewage and 1 × 10^−1^ CFU/100 mL for recycled water.

### DNA extraction for microbial analysis

For all surface water samples, 500 mL of each water sample was filtered through a Fisherbrand 47 mm mixed cellulose ester membrane with 0.45 µm pores. Membrane filters were aseptically folded and placed in Qiagen PowerBead Tubes and stored at −80°C (<1 month). Untreated sewage samples (10 mL) were mixed with 990 mL of PBS (pH 7.4) in a sterile beaker for 1 min with a magnetic stirrer and then concentrated by membrane filtration as described above. Recycled water was directly filtered to concentrate 1 L by membrane filtration as described above. DNA extractions were performed on all water samples using the Qiagen DNeasy PowerWater Kit using the manufacturer’s instructions. qPCR for MST markers was performed as described in the section below. One hundred microliters of purified DNA was eluted for all samples in this study.

### qPCR analysis of microbial variables

qPCR was conducted to quantify sewage-associated *Bacteroides* HF183 following USEPA method 1696 (7) in surface water and WWTF samples. A general *E. coli* target EC23S857 (37), sewage-associated H8 (35), and crAssphage CPQ_056 (38) were also tested on DNA extracted from AWT and CWT facilities. qPCR amplification was conducted in 25 µL reactions in triplicate using 12.5 µL TaqMan Environmental Master Mix 2.0 (Applied Biosystems) and 5 µL of template DNA per reaction in a Bio-Rad CFX96 Touch Real-Time PCR Detection System (Bio-Rad Laboratories, CA, USA). All assays included 40 thermal cycles, and primer and probe concentrations/sequences and qPCR assay conditions are reported in Table S1. Standard curves were constructed from synthetic gene fragments (gBlocks, Integrated DNA Technologies) (Table S2) containing the target sequences, and reference DNA material (inhibition amplification control, HF183 only) was included for each sample according to the guidelines in USEPA method 1696 (7). All standard curves ranged from 10^7^ to 5 gene copies per reaction. Performance metrics included efficiencies between 90% and 110%, and *R*^2^ values ranging from 0.979 for EC23S857 to 0.998 for HF183. In untreated sewage samples, the limit of detection was 500 GC/100 mL, and the limit of quantification was 1,000 GC/100 mL for each qPCR assay. For recycled water samples, the limit of detection was 10 GC/100 mL, and the limit of quantification was 20 GC/100 mL for each qPCR assay. Inhibition of qPCR amplification was not detected in any of the samples tested in this study (data not shown). Negative controls for each instrument run included four extraction blanks and four non-template controls, which were all negative for each qPCR target in this study.

### Molecular analysis of culturable EcH8

Isolated colonies with characteristic *E. coli* morphology were individually picked with a sterile toothpick from mTEC agar plates for culturable EcH8 analysis. Each colony was suspended in 50  µL of reagent-grade water and boiled for 10 min at 100°C in a thermal cycler as described in a previous study (35). PCR amplification of an *E. coli-*specific β-glucuronidase *uid*A gene was conducted to confirm colonies as *E. coli* (39). Confirmed colonies (30 per sample) extracted by boiling lysis were also individually tested for the presence of the H8 gene by PCR. PCR amplification was conducted in 25 µL reactions that included 12.5 µL TaqMan Environmental Master Mix 2.0 (Applied Biosystems) and 5 µL of template DNA per reaction in a Bio-Rad CFX96 Touch Real-Time PCR Detection System (Bio-Rad Laboratories, CA, USA). Primer and probe concentrations/sequences and PCR assay conditions are reported in Table S1. A genomic positive control for *uid*A and H8, ATCC 13706 was included in each run for comparison. An H8 negative control (ATCC 11775) was also included in each run.

### Data analyses

R version 4.1.3 (40) was used for statistical analyses in this study, which included descriptive statistics, hypothesis testing, and correlation analyses. A significance threshold of *α* < 0.05 was set for all statistical tests. LOQ was defined as the lowest standard concentration that consistently amplified in all three replicates, while LOD was the lowest standard concentration in which at least two out of three replicates consistently amplified. Data that were below the LOQ and the LOD were considered censored, and values were calculated for statistical analyses (41). In this study, left-censored data were only in recycled water samples, and censored observations were only below the LOD. These data were expressed as a range from 0 to LOD – 1. To account for censored observations in microbial data, *u*-scores were calculated based on version 1.2 of *u*-score script written by Helsel (available in PracticalStats.com) (41). *u*-scores were utilized to compute statistical analyses that tested for relationships between microbial variables and differences by treatment type. All data were log_10_-transformed prior to statistical analysis.

Descriptive statistics (median and standard deviation) were calculated by treatment type (AWT or CWT) and sample type (untreated sewage or recycled water) for culturable *E. coli* and qPCR (EC23S857, HF183, and H8, CPQ_056) data through the R package stats (version 4.1.3). The nonparametric Kruskal-Wallis one-way analysis of variance by rank test was executed for univariate analysis of significant differences in microbial variables in untreated sewage and recycled water from AWT compared to CWT facilities. Differences among microbial variables were determined by Kruskal-Wallis rank test followed by pairwise comparisons using Dunn’s test for multiple comparisons in untreated sewage and recycled water data through the R package rstatix (version 0.7.2), with the Benjamini and Hochberg method used for *P*-value correction. Left-censored microbial data for recycled water samples included qPCR-derived measurements (*n* = 18) of HF183 (22%), H8 (33%), and CPQ_056 (22%). Robust regression on order statistics was implemented via the R package NADA (version 1.6-1.1) to compute medians and interquartile ranges for left-censored data (41, 42). The frequency of detection was determined for each microbial variable and compared across treatment type (AWT and CWT) in untreated sewage or recycled water data sets with a Fisher’s exact test (43) and the R package rstatix (version 0.7.2), with the Benjamini and Hochberg method used for *P*-value correction. Proportions of culturable *E. coli* with the H8 gene (culturable EcH8) were compared across wastewater treatment facilities, and significant differences were determined through Fisher’s exact test (43). Relationships between microbial variables were analyzed with Kendall’s rank correlation tau, where the coefficient (tau) can range from −1.0 to 1.0. A value of −1.0 designates a perfect negative correlation between two variables, and a value of 1.0 indicates a perfect positive correlation. A value of 0 demonstrates that no linear relationship exists between two variables.

#### Effects of advanced vs conventional treatment on concentrations of microbial variables

A two-way permutational multivariate analysis of variance (PERMANOVA) was executed with the vegan R package (44) to determine if there was a significant effect of treatment type (AWT or CWT) on all concentrations of fecal (EC23S857) and sewage indicators measured (HF183, H8, and CPQ_056) in recycled water. The homogeneity of the multivariate dispersion assumption was met prior to PERMANOVA among groups (treatment type). Linear discriminant analysis (LDA) was performed to visualize the variability in measurements among AWT and CWT facilities. LDA was accomplished by constructing an ordination plot through a canonical analysis of principal coordinates via the Biodiversity R package (45).

#### Analysis of the relationship between HF183 and culturable EcH8 in surface waters

HF183 concentrations were log_10_ transformed and compared to the frequency of culturable EcH8 detection by binary logistic regression. Detection of culturable EcH8 was defined as 1.0 if there was amplification of DNA from at least 1 isolate out of the 30 tested for a given sample, or 0.0 was assigned if 0 out of 30 isolates were amplified for culturable EcH8. Binary logistic regression was carried out using GraphPad Prism version 10.0.2. to determine log-likelihood, odds ratio, and the confidence interval around the odds ratio.

## RESULTS

### Discharge study: impact of recycled water on HF183 concentrations in a Florida stream

A Florida stream (Fig. S1) that receives 1–6 million gallons of recycled water during sporadic discharge events was sampled to measure HF183 concentrations prior to discharge, during a known discharge event, and after discharge (Table 2). The stream on average is approximately 5.15 meters wide and 0.4 meters deep with an average flow of 0.26 meters/s. HF183 was detected at the discharge outfall site at low concentrations (1.3 log_10_ GC/100 mL) prior to the discharge and was neither detected at the upstream site nor the downstream site (Table 2). During discharge, HF183 concentrations increased by approximately three orders of magnitude in the discharge outfall (4.15 log_10_ GC/100 mL) and at the downstream site (3.58 log_10_ GC/100 mL), while HF183 was not detected in the site upstream of the outfall (Table 2). After discharge, HF183 was only detected at the downstream site and was about two orders of magnitude lower than what was recorded during the recycled water discharge event (1.63 log_10_ GC/100 mL) (Table 2). During effluent discharge, concentrations of culturable *E. coli* at the discharge site were reduced to <1 CFU/100 mL and were about 3.31 log_10_ CFU/100 mL at the downstream site, but the concentrations at both sites returned to their previous concentration the next day (Table 2).

**TABLE 2:** HF183 and culturable *E. coli* concentrations (log_10_ GC or CFU/100 mL) in a stream that receives recycled water from an AWT facility^*c*^

Temporal relationship to discharge	Upstream site (log_10_ CFU or GC/100 mL)		Recycled water discharge outfall (log_10_ CFU or GC/100 mL)		Downstream site (log_10_ CFU or GC/100 mL)	
	*E. coli*	HF183	*E. coli*	HF183	*E. coli*	HF183
Prior to discharge	2.88	<LOD^*a*^	2.54	1.30	2.30	<LOD
During discharge	2.00	<LOD	<LOD	4.15	3.31	3.58
After discharge^*b*^	1.88	<LOD	1.95	<LOD	2.43	1.63

^*a*^ <LOD, below qPCR limit of detection.

^*b*^ Sampled 23 hours after discharge was discontinued.

^*c*^ The stream was sampled upstream of the outfall, at the outfall, and downstream before, during, and after discharge of recycled water.

### Association of facility type (AWT and CWT) with microbial variables in untreated sewage and recycled water

**Fig 1:**
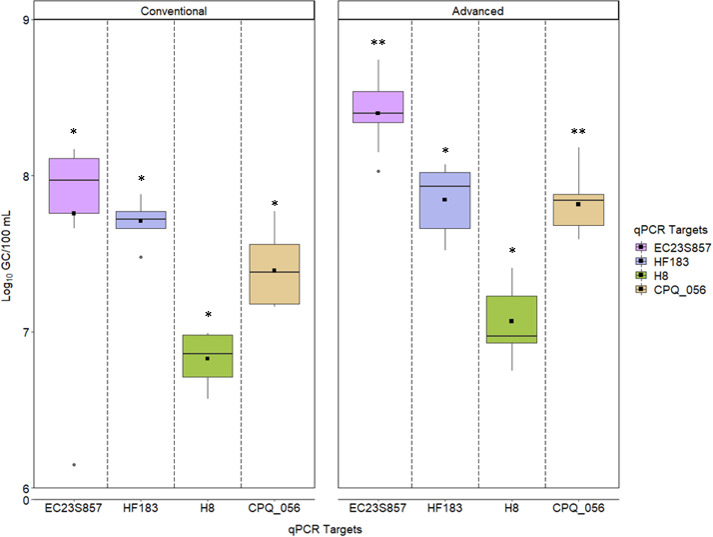
Concentrations of microbial variables (log_10_ GC/100 mL) in untreated sewage from advanced and conventional facilities. The interquartile range (25th and 75th percentile) includes the log_10_ medians (black horizontal bar) and means (black square) for each qPCR marker across conventional and advanced WWTFs. Boxplot whiskers represent the 10th and 90th percentile values. Outliers are displayed as black dots above or below each boxplot, the *y*-axis was truncated to show data ranging from 6.0 to 9.0 log_10_ GC/100 mL. Each plant was sampled on three separate events. Asterisks denote a comparison of values between conventional and advanced WWTFs. Variables with different numbers of asterisks across the WWTF types are significantly different (*α* = 0.05), e.g., EC23S857 is significantly greater in AWT compared to CWT facilities.

We compared microbial variables by qPCR in untreated sewage and recycled water from AWT vs CWT facilities. For untreated sewage, the concentration and frequency of detection of microbial variables were measured. Concentrations of crAssphage (CPQ_056) and the general *E. coli* marker gene EC23S857 were significantly greater in untreated sewage from AWT compared to CWT facilities, while HF183 and H8 concentrations were not significantly different (Fig. 1, *P*-values in Table S3). Multivariate analysis of all microbial variables showed that concentrations were significantly greater in untreated sewage collected from AWT facilities compared to CWT facilities (Fig. S2). All microbial variables were quantifiable in each untreated sewage sample from AWT and CWT facilities.

In recycled water samples, we compared the concentrations of qPCR marker genes and their persistence (frequency of detection or log_10_ reduction) between AWT and CWT facilities.

**Fig 2:**
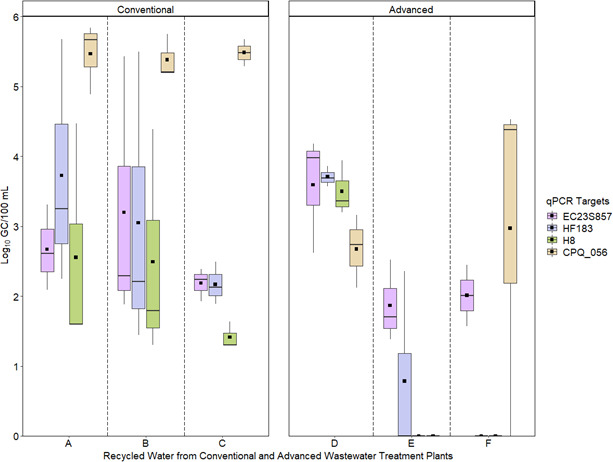
Concentrations of microbial variables (log_10_ GC/100 mL) in recycled water from AWT and CWT facilities. The interquartile range (25th and 75th percentile) includes the log_10_ medians (black horizontal bar) and means (black square) for each marker across conventional and advanced WWTFs. Boxplot whiskers represent the 10th and 90th percentile values. Each plant was sampled on three separate events.

**Fig 3:**
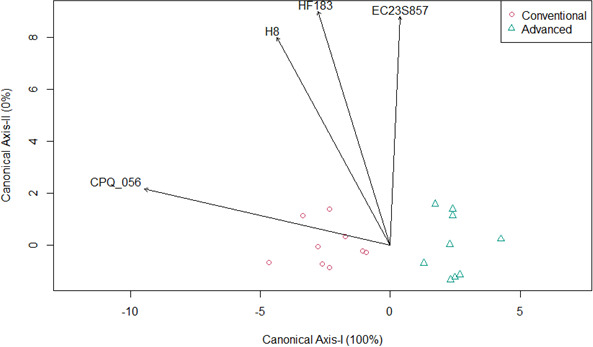
Relationships among microbial variables measured by qPCR in recycled water produced by AWT and CWT facilities (conventional, red circles and advanced, green triangles) analyzed by canonical analysis of principal coordinates and linear discriminant analysis. Canonical axis I (horizontal) explained 100% of the variability, while canonical axis II (vertical) explained 0% of the variability observed.

Concentrations of crAssphage CPQ_056 and the H8 marker (2.12 and 0.99 log_10_ GC/100 mL, respectively) were significantly lower in recycled water produced by AWT facilities compared to CWT facilities (5.67 and 1.52 log_10_ GC/100 mL, respectively), while no significant difference was observed for EC23S857 and HF183 (Fig. 2, *P*-values in Table 3). Canonical analysis of principal coordinates demonstrated a clear separation of microbial variables in recycled water produced in AWT compared to CWT facilities (Fig. 3). CPQ_056 and the H8 marker were the variables that best explained differences in concentrations of microbial variables in recycled water produced by AWT and CWT facilities (Fig. 3). HF183 and H8 markers were significantly less frequently detected in recycled water from AWT facilities (44% and 33%, respectively) compared to CWT facilities (100%) (Tables 3 and 4), while crAssphage CPQ_056 showed a similar trend but was not significant (*P* = 0.0824; Table 3). Multivariate analysis of all microbial variables showed that concentrations were significantly greater in recycled water from CWT facilities compared to AWT facilities (Table 3).

**TABLE 3:** Statistical comparisons of microbial variables measured by qPCR in recycled water produced in AWT and CWT facilities^*d*^^*,^e^*^

qPCR variables	*P* value: differences among median log_10_ concentrations	*P* value: differences among log_10_ reduction	*P* value: differences in frequency of detection
Univariate			
EC23S857^*a*^^,*c*^	0.7573	0.2004	1.0000
HF183^*a*^^,*c*^	0.1957	0.2332	**0.0294**
H8^*a*^^,*c*^	**0.0218**	0.1451	**0.0091**
CPQ_056^*a*^^,*c*^	**0.0003**	**0.0004**	0.0824
Multivariate: all^*b*^^,*c*^ (advanced vs conventional)	**0.0120**	**0.0110**	**<0.0001**

^*a*^ Variables were individually compared between AWT and CWT facilities by Kruskal-Wallis rank sum tests.

^*b*^ Variables were compared as a group by permutational multivariate analysis of variance.

^*c*^ Differences in the frequency of detection were compared by Fisher’s exact test.

^*d*^ *P*-values < 0.05 are bolded.

^*e*^ Data are expressed as concentration (log_10_ GC/100 mL), log_10_ reduction, and frequency of detection. Data from like facilities (AWT or CWT) were pooled (*n* = 9)*.*

**TABLE 4:** Frequency of detection of microbial variables (general fecal and sewage-associated MST markers) measured by qPCR in recycled water from advanced and conventional wastewater treatment plants^*a*^

Facility	Frequency of detection (%)			
	EC23S857	HF183	H8	CPQ_056
Conventional				
A	100	100	100	100
B	100	100	100	100
C	100	100	100	100
CWT data combined (*n* = 9)	100	100	100	100
Advanced				
D	100	100	100	100
E	100	33	0	0
F	100	0	0	67
AWT data combined (*n* = 9)	100	44	33	56
AWT and CWT				
Data pooled (*n* = 18)	100	72	67	78

^*a*^ Each plant was sampled on three separate events.

**Fig 4:**
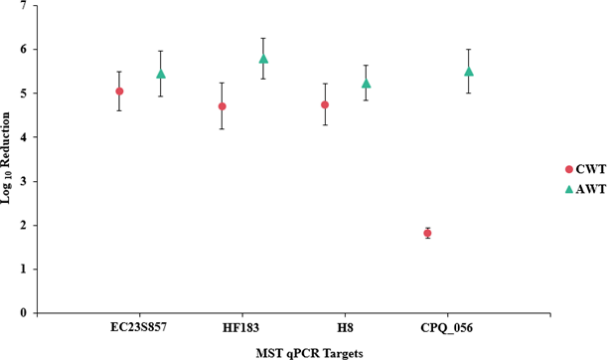
Mean log_10_ reduction (decay) of microbial variables measured by qPCR in recycled water from AWT and CWT wastewater treatment facilities. Error bars are standard error of mean log_10_ reduction values.

The persistence of each microbial variable was further explored by comparing decay rates (log_10_ reduction) from untreated sewage to recycled water in AWT compared to CWT facilities, as this metric accounts for initial concentration in influent as well as final concentration in recycled water. Univariate analysis showed that decay rates of all microbial variables were somewhat greater in AWT facilities compared to conventional treatment (Fig. 4), although only CPQ_056 experienced significantly greater log_10_ reduction of 5.50 in AWT facilities compared to 1.82 in CWT facilities (Fig. 4; Table 3). For all microbial variables, the multivariate analysis showed a significantly lower persistence (frequency of detection and log_10_ reduction) in AWT compared to CWT facilities (Table 3). All log_10_ reduction values measured in this study are available in Table S4.

### Differences among microbial variables: pooled data from AWT and CWT facilities

We pooled data from all facilities to focus on differences among concentrations of microbial variables irrespective of treatment strategies. In untreated sewage, all microbial variables measured by qPCR were detected and quantifiable in each untreated sewage sample tested during this study. *E. coli* EC23S857 marker concentrations (8.14 log_10_ GC/100 mL) were the highest followed by HF183 (7.76 log_10_ GC/100 mL), crAssphage CPQ_056 (7.63 log_10_ GC/100 mL), and H8 marker (6.95 log_10_ GC/100 mL) (Fig. 1). EC23S857 marker concentrations were significantly greater than all other variables except HF183 (*P*-values in Table S5), although the comparison was on the verge of significance (*P* = 0.0512). The concentrations of all other microbial variables were significantly greater than the H8 marker (Table S5). HF183 and crAssphage CPQ_056 concentrations were not significantly different in pooled untreated sewage data (Table S5).

In recycled water, we examined which markers were most dominant and prevalent. We pooled data on microbial variables measured by qPCR and compared differences among microbial variables using three metrics: concentration, frequency of detection, and log_10_ reduction. The median concentration of CPQ_056 (4.71 log_10_ GC/100 mL) was ranked the highest followed by EC23S857 (2.42 log_10_ GC/100 mL), HF183 (2.23 log_10_ GC/100 mL), and H8 marker (1.26 log_10_ GC/100 mL). CPQ_056 concentrations were significantly greater than that of H8 marker but were not significantly different from HF183 or EC23S857 (Table S5). No other comparisons of median values in recycled water were significant (Table S5). A comparison of the frequency of detection (Table 4) found that EC23S857 (100%) was significantly greater than HF183 (72%) and the H8 marker (67%) in pooled recycled water data (Table S5). CPQ_056 frequency of detection (78%, Table 4) was not significantly different from that of any of the bacterial variables (EC23S857, HF183, and H8) (Table S5). Median log_10_ reduction values (Table S4) (~2.8–5.76 log_10_) were not significantly different among qPCR marker genes when all recycled water data were pooled (Table S5).

We also examined relationships among microbial variables measured by qPCR in untreated sewage or recycled water to compare differences in their removal in pooled data sets from AWT and CWT facilities. Significant relationships among microbial variables were found in untreated sewage data, i.e., concentrations of the H8 marker were positively correlated with EC23S857, HF183, and crAssphage CPQ_056, while EC23S857 levels positively correlated with CPQ_056 concentrations (Fig. S3, *P*-values in Table S6). HF183 concentrations were not correlated with the levels of EC23S857 or CPQ_056 (Table S6). In recycled water, significant positive correlations were found among concentrations of all bacterial variables (HF183, H8 marker, and EC23S857) (Fig. S4; Table S6). No relationship was found between CPQ_056 concentrations and any bacterial variables (EC23S857, HF183, and H8 marker) in all recycled water data (Fig. S4; Table S6).

### Culturable *E. coli* and culturable EcH8 in untreated sewage and recycled water

Concentrations of culturable *E. coli* and the proportion carrying the H8 gene were compared across AWT and CWT facilities in untreated sewage and recycled water samples. Culturable *E. coli* concentrations in untreated sewage were not significantly different among all WWTFs, ranging from 6.46 to 6.67 log_10_ CFU/100 mL (Table 5; Table S3). Culturable EcH8 was detected in all untreated sewage samples, and estimated concentrations of culturable EcH8 obtained by multiplying total *E. coli* concentration by the percentage of colonies positive for H8 ranged from 5.24 to 6.02 log_10_ CFU/100 mL. Culturable EcH8 in untreated sewage comprised ~14% of *E. coli* colonies tested over the duration of the study. The frequency of culturable EcH8 in the culturable *E. coli* population ranged from 8% to 18% at the various WWTFs and was not significantly different in untreated sewage from AWT compared to CWT facilities (Table 5; Table S3). No *E. coli* were detected in recycled water samples (<0.1 CFU/100 mL, *n* = 18) over the duration of the study; therefore, no culturable *E. coli* could be tested for the H8 gene in recycled water.

**TABLE 5:** Mean culturable *E. coli* concentrations (log_10_ CFU/100 mL ± standard error) and the proportion of tested colonies positive for the H8 gene (culturable EcH8) in untreated sewage from AWT and CWT plants^*a*^

Facility	Culturable *E. coli* (log_10_ CFU 100 mL^−1^)	Colonies tested for EcH8	Proportion EcH8-positive (%)
Conventional WWTF untreated sewage			
A	6.67 ± 0.04	90	17.7 ± 3.9
B	6.59 ± 0.04	90	15.7 ± 1.3
C	6.52 ± 0.12	90	15.7 ± 4.3
Advanced WWTF sewage			
D	6.56 ± 0.05	90	9.0 ± 2.0
E	6.46 ± 0.03	90	8.0 ± 1.0
F	6.47 ± 0.03	90	17.0 ± 5.8

^*a*^ Each plant was sampled on three separate events.

### HF183 and culturable EcH8 relationship in surface water survey

**Fig 5:**
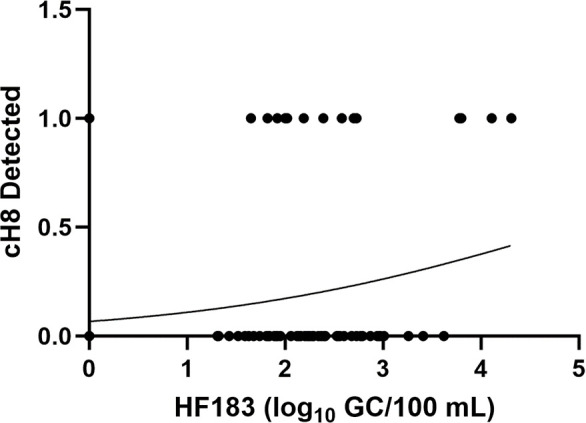
Binary logistic regression of a significant positive relationship between culturable EcH8 detection and HF183 concentrations (log_10_ GC/100 mL) in contaminated surface water samples (*n* = 103).

Surface water samples from the St. Petersburg area characterized by chronically elevated fecal indicator bacteria levels contained HF183 levels ranging from below LOD to 4.31 log_10_ GC/100 mL. Culturable EcH8 was detected in 16.5% of 103 surface water samples (Table S7). HF183 was detected in 82.5% of these samples and in 94.1% of the 17 samples that were positive for culturable EcH8 (Table S7). HF183 concentration and culturable EcH8 detection were positively and significantly correlated by logistic regression (*P* = 0.0354) (Fig. 5). The odds ratio of the logistic regression was β_1_ = 1.701 with a 95% confidence interval of 1.068–2.921.

## DISCUSSION

Recycled water production for urban, industrial, environmental, and agricultural applications in the United States is estimated to rise from 4.8 to 6.6 billion gallons per day by 2027 according to a Bluefield research survey (46). Recycled water delivered to surface waters can be misidentified as untreated sewage due to the persistence of sewage-associated markers such as HF183 and crAssphage (CPQ_056) through wastewater treatment (22, 47, 48). The persistence of other sewage-associated MST markers through wastewater treatment and in environmental waters is less understood, and a comparative study of MST marker persistence in AWT and CWT facilities has, to our knowledge, not previously been described. We have noted the potential for the influence of recycled water on the occurrence of MST markers associated with untreated sewage at many sites in Florida and were able to directly demonstrate it in a discharge event from an AWT facility described in this study. This work has advanced the MST field by showing that the persistence of sewage-associated markers was dependent on the treatment level and was shown to be significantly lower in AWT vs CWT recycled water by multivariate analysis. It also provides novel data that demonstrate the effect of recycled water discharge from an AWT facility on MST markers in surface water and the use of culturable EcH8 in viable *E. coli* to discriminate between untreated sewage and recycled water.

### Persistence and levels of sewage-associated MST marker genes in AWT and CWT facilities

Multiple environmental and experimental design factors influence the observed variability of microorganisms and their genes in treated wastewater effluent (reviewed in reference 49). Variable persistence of microbes and their DNA can be influenced by factors such as treatment strategy utilized (50), air temperature, and elevation (51). Within a facility, the time of the day the sample is collected (52), flow rate (53), and seasonal effects (54) can all influence microbial concentrations. Post-secondary treatment stages in AWT facilities sampled here include anoxic basins and oxidation ditches, dual media deep-bed denitrification filters, activated sludge treatment in an anoxic/aerobic configuration, and a five-stage Bardenpho activated sludge process, each of which could feasibly contribute to the reduction of DNA in recycled water. All facilities in this study utilized chlorination for disinfection except AWT facility D, which only used ultraviolet light for disinfection. AWT facility D performed poorly in terms of DNA reduction and was also the only AWT facility with recycled water that contained detectable and quantifiable levels of all qPCR markers for each sample tested in this study. High concentrations of HF183 and the H8 marker in recycled water produced by AWT facility D led to non-significant differences in log_10_ reduction of MST markers between AWT and CWT facilities. In general, differences among WWTFs complicate generalizations about treatment efficacy, and the issue is exacerbated in AWT facilities, where many treatment options exist (e.g., Table 1). Studies of microbial persistence through wastewater treatment should therefore include salient details about treatment processes to maximize the usefulness of the data.

Multivariate and univariate analyses of microbial variables in AWT and CWT facilities showed lower concentrations and greater persistence of most microbial variables measured by qPCR in AWT facilities. Univariate analysis of individual variables revealed significantly lower levels and greater decay of crAssphage CPQ_056 in AWT compared to CWT facilities by all metrics; in fact, CPQ_056 log_10_ reduction in CWT recycled water was only ~2 log_10_, compared to ~5.5 log_10_ in AWT recycled water. To the best of our knowledge, only one other study has reported qPCR measurements of a sewage-associated indicator in recycled water produced by AWT and CWT facilities. Morrison et al. (55) found that the frequency of detection of CPQ_056 was 43% in recycled water produced in an AWT facility compared to 76% from a CWT facility in Arizona (USA). These findings agree with the data from this study; however, any broad generalizations must await further studies.

Several studies report MST marker persistence in WWTFs, measuring DNA targets such as HF183 and crAssphage (e.g., CPQ_056); however, they did not compare data from AWT compared to CWT facilities (22, 56, 57). Several studies conducted in the United States measured HF183 in disinfected effluent, finding less effective reduction compared to our study (58, 59). Two AWT facilities sampled in the U.S. produced recycled water containing HF183 in 100% of samples, and log_10_ reduction values ranged from 2.0 to 4.2 (58), whereas HF183 in recycled water from AWT facilities in our study was detected in only 44% of samples, with log_10_ reduction values from 4.1 to 6.9. Removal of HF183 in recycled water from a CWT facility was two orders of magnitude lower than CWT facilities tested in our study (~3 compared to ~5 log_10_ reduction [59]). This study (59) used only 100 mL sample sizes and acidified the sample prior to filtration, which may have lowered the recovery of the bacterial HF183 gene target. Another study conducted in northeast England detected HF183 and HumM2 (sewage-associated genetic marker) in 100% of disinfected effluent samples, and median log_10_ reductions ranged from 1.3 to 1.4 for the 15 WWTFs tested; however, this study included 12 small treatment facilities and did not provide AWT or CWT classification (54). These few papers form the probing edge of our knowledge about the persistence of sewage-associated markers in AWT and CWT facilities. What is clear is that bacterial and viral MST marker DNA persists through AWT and CWT facilities in the United States and in other countries, which could confound the identification of untreated sewage in surface waters when recycled water is present.

### Relative persistence of sewage-associated MST markers through wastewater treatment

Very few comparative studies of the persistence of bacterial and viral MST marker DNA in recycled water have been performed. We found that crAssphage CPQ_056 was markedly more persistent than other microbial variables measured in this study, particularly in CWT facilities, where we observed ~2 log_10_ reduction, in contrast to ~5 log_10_ reduction of the bacterial variables measured by qPCR. Furthermore, no relationship between CPQ_056 concentrations and those of any bacterial variable was observed in recycled water; however, bacterial variables were all correlated, suggesting different drivers of decay between viral and bacterial targets. A study conducted at a CWT facility (Indiana, USA) found similar persistence of CPQ_056 (2.88 log_10_ reduction) compared to HF183 (3.33 log_10_ reduction) in disinfected effluent, in contrast to our study; however, only one facility was sampled (59). Other sewage indicators such as human adenovirus (HAdV) and human polyomaviruses (HPyVs) were significantly more persistent than HF183 and CPQ_056 in a U.S. study, with mean log_10_ reductions of 3.33 (HF183), 2.88 (CPQ_056), 2.24 (HAdV), and 1.51 (HPyVs) in disinfected effluent (59). These few studies demonstrate that the persistence of sewage indicators varies in different wastewater treatment facilities and that the ability to distinguish between treated and untreated sewage in surface water applications could represent a major advance.

Differential persistence of sewage-associated microbes through wastewater treatment was noted in this study. Variability in the persistence of bacteria can be attributed in part to cellular physiology, e.g., tolerance to low nutrients and temperature (60). However, DNA from sewage-associated viruses tends to display greater persistence than bacterial DNA through wastewater treatment (22, 57), which can be explained in part by size; viruses are smaller than bacteria and are not consistently removed in settling tanks (reviewed in reference 61). Furthermore, viruses are generally not as susceptible as bacteria to chemical disinfectants (62, 63). One challenge in utilizing viral markers (e.g., HAdV and HPyVs) to estimate the persistence of viruses is that viruses are generally several orders of magnitude lower than concentrations of HF183 in untreated sewage and may not be detectable in diluted effluents (64). CrAssphage DNA, on the other hand, is present at higher concentrations than other viral marker genes in untreated sewage and was more persistent through wastewater treatment than any bacterial variable in this study, which further supports its use as an indicator for viral persistence in WWTFs. Understanding the differential persistence of MST marker genes through wastewater treatment and their concentration in disinfected effluent or recycled water will further support the selection of optimal markers for different research and regulatory applications.

### H8 in culturable *E coli:* confirmation of untreated sewage contamination when recycled water is a confounding factor

This study has shown that testing DNA extracted from surface waters for H8 via qPCR is fraught with the same issues as other qPCR assays, i.e., DNA persists through wastewater treatment and presents the same limitations as other methods such as HF183 and crAssphage. The H8 sequence has also been reported in *Klebsiella* and *Yersinia* strains that are not associated with the human gastrointestinal tract (34, 65); hence, optimal strategies should involve cultivation and isolation of *E. coli* prior to H8 testing. Accurate identification of untreated sewage in surface waters that are also impacted by recycled water can be supported by a confirmatory step to avoid positive indications of untreated sewage and overestimation of health risks that can arise from sole reliance on nucleic acid-based methods. Utilizing a method that relies on the detection of viable bacteria that are also associated with human feces and untreated sewage is one possible path. Because culturable *E. coli* are at such low levels in recycled water that they are rarely detected (this study; 20, 66), amplification of the sewage-associated H8 gene in viable *E. coli* (culturable EcH8) can be applied to circumvent the limitations inherent in qPCR-based testing for untreated sewage when treated wastewater is present. The surface water survey in this study found a significant positive relationship between culturable EcH8 detection and HF183 concentration in water bodies impacted by untreated sewage and recycled water around Tampa Bay, FL, USA.

Another advantageous characteristic of the culturable EcH8 assay is the widespread use of *E. coli* as a fecal indicator for contamination of water and food (35, 67). Culturable *E. coli* is a commonly used regulatory tool for the assessment of recreational water quality (36) and many other aspects of sanitation, including wastewater treatment; therefore, many facilities in the U.nited States and other countries have the capacity to quantify *E. coli* by culture methods. The culturable EcH8 method in viable *E. coli* can also be utilized to identify the presence of human fecal contamination in other applications such as household or food industry studies that test surfaces and food to estimate human health risks.

The proportion (8%–18% in this study) of culturable *E. coli* with H8 in untreated sewage varied across WWTFs, suggesting the need to test untreated sewage at facilities near a site of interest prior to embarking upon a study. The specificity of culturable EcH8 for human/sewage sources in previous studies ranged from 92% to 99% in Australia (65), Japan (34), Thailand (68), and the U.nited States (35). Culturable EcH8 was detected in all untreated sewage samples in this study and a previous U.S. study (35). Lower sensitivity was reported when reference samples included individual fecal samples, i.e., in Australia at 45% (65), Japan at 30% (34), and Thailand at 36% (68), suggesting that the culturable EcH8 genetic marker is not shed in all individuals. Variable shedding in individuals is common in sewage-associated MST markers such as the HF183 marker (69), and person-to-person variability for the gut microbiome has been well documented in the literature (70–72). Variability in culturable EcH8 performance could occur across geographical regions, and parameters such as sensitivity and specificity should be evaluated in new study locations with local fecal/untreated sewage samples. Recycled water applications and usage vary widely across the U.nited States and in other countries, affecting the potential of recycled water to confound surface water quality testing; therefore, knowledge of irrigation and other practices that may deliver recycled water or treated wastewater to surface waters is necessary to maximize interpretation of MST results.

Future directions for the culturable EcH8 method of detecting viable sewage-associated *E. coli* should explore strategies to increase the number of *E. coli* isolates tested. Testing more *E. coli* colonies will improve method sensitivity, while extracting DNA from composite samples made from multiple colonies can improve workflow and adaptability of this approach. Coupled with a standard MST marker that targets untreated sewage, e.g., HF183 and culturable EcH8, can be used as a confirmation step for the identification of untreated sewage contamination in surface waters and will improve MST interpretations in water bodies that receive substantial inputs of recycled water.

In summary, this study showed that DNA in recycled water released from an AWT facility could raise HF183 levels 1,000-fold in receiving waters. We demonstrated the persistence of *E. coli* and MST marker DNA through AWT and CWT in six WWTFs, supporting the premise that DNA released in recycled water and other disinfected effluent can cause a false-positive indication of untreated sewage contamination in environmental waters. The comparison of DNA removal through wastewater treatment in facilities with different levels of treatment showed that AWT facilities were more effective than CWT facilities at removing DNA, particularly in the case of crAssphage CPQ_056. We demonstrated that culturable EcH8 has a strong potential to discriminate between the presence of untreated sewage compared to disinfected effluent in environmental waters by demonstrating undetectable levels of cultured *E. coli* in recycled water, and thus the absence of EcH8 in culturable *E. coli* isolates, and by showing that culturable EcH8 can be detected in contaminated surface waters and that its detection is correlated with HF183 concentrations. The usefulness of culturable EcH8 to discriminate untreated sewage from recycled water sources can be improved by modifying the method to screen more *E. coli* colonies per sample, thus increasing sensitivity. It should also be further explored by measuring the persistence of culturable EcH8 through varying wastewater treatment systems and how it persists in surface waters exposed to environmental conditions.

## Data Availability

Data are in the University of South Florida (USF) Digital Commons (https://digitalcommons.usf.edu) at DOI 10.17632/5dh58k5x2f.1.

## SUPPLEMENTAL MATERIAL

Supplemental material mbio.00655-24-s0001.pdf Fig. S1 to S4; Tables S1 to S7.

ASM does not own the copyrights to Supplemental Material that may be linked to, or accessed through, an article. The authors have granted ASM a non-exclusive, world-wide license to publish the Supplemental Material files. Please contact the corresponding author directly for reuse.

## References

[1] Cheung WHS, Chang KCK, Hung RPS, Kleevens JWL. 1990. Health effects of beach water pollution in Hong Kong. Epidemiol Infect 105:139–162. doi:10.1017/S0950268800047737 2384140 PMC2271797

[2] Prüss A. 1998. Review of epidemiological studies on health effects from exposure to recreational water. Int J Epidemiol 27:1–9. doi:10.1093/ije/27.1.1 9563686

[3] Wade TJ, Pai N, Eisenberg JNS, Colford JM Jr. 2003. Do US environmental protection agency water quality guidelines for recreational waters prevent gastrointestinal illness? A systematic review and meta-analysis. Environ Health Perspect 111:1102–1109. doi:10.1289/ehp.6241 12826481 PMC1241558

[4] Soller JA, Bartrand T, Ashbolt NJ, Ravenscroft J, Wade TJ. 2010. Estimating the primary etiologic agents in recreational freshwaters impacted by human sources of faecal contamination. Water Res 44:4736–4747. doi:10.1016/j.watres.2010.07.064 20728915

[5] Soller JA, Schoen ME, Bartrand T, Ravenscroft JE, Ashbolt NJ. 2010. Estimated human health risks from exposure to recreational waters impacted by human and non-human sources of faecal contamination. Water Res 44:4674–4691. doi:10.1016/j.watres.2010.06.049 20656314

[6] Harwood VJ, Staley C, Badgley BD, Borges K, Korajkic A. 2014. Microbial source tracking markers for detection of fecal contamination in environmental waters: relationships between pathogens and human health outcomes. FEMS Microbiol Rev 38:1–40. doi:10.1111/1574-6976.12031 23815638

[7] Shanks O, Sivaganesan M, Kelty C, Haugland R. 2019. Method 1696: characterization of human fecal pollution in water by HF183/BacR287 TaqMan quantitative polymerase chain reaction (qPCR) assay. Office of Water, Washington, DC.

[8] McCaffrey DF, Sloss EM, Fricker RD, Ritz BR, Geschwind SA. 1999. Groundwater recharge with reclaimed water: birth outcomes in Los Angeles County, 1982-1993.

[9] Nellor MH, Baird RB, Smyth JR. 1985. Health effects of indirect potable water reuse. Journal AWWA 77:88–96. doi:10.1002/j.1551-8833.1985.tb05573.x

[10] Ritz BR, Geschwind SA, McCaffrey DF, Sloss EM. 1996. Groundwater recharge with reclaimed water: an epidemiologic assessment in Los Angeles County, 1987-1991.

[11] Sinclair M, O’Toole J, Forbes A, Carr D, Leder K. 2010. Health status of residents of an urban dual reticulation system. Int J Epidemiol 39:1667–1675. doi:10.1093/ije/dyq152 20817699

[12] Wade TJ, Calderon RL, Brenner KP, Sams E, Beach M, Haugland R, Wymer L, Dufour AP. 2008. High sensitivity of children to swimming-associated gastrointestinal illness: results using a rapid assay of recreational water quality. Epidemiology 19:375–383. doi:10.1097/EDE.0b013e318169cc87 18379427

[13] Betancourt WQ, Duarte DC, Vásquez RC, Gurian PL. 2014. Cryptosporidium and Giardia in tropical recreational marine waters contaminated with domestic sewage: estimation of bathing-associated disease risks. Mar Pollut Bull 85:268–273. doi:10.1016/j.marpolbul.2014.05.059 24975093

[14] Fleisher JM, Kay D, Wyer MD, Godfree AF. 1998. Estimates of the severity of illnesses associated with bathing in marine recreational waters contaminated with domestic sewage. Int J Epidemiol 27:722–726. doi:10.1093/ije/27.4.722 9758131

[15] Nappier SP, Soller JA, Eftim SE. 2018. Potable water reuse: what are the microbiological risks? Curr Environ Health Rep 5:283–292. doi:10.1007/s40572-018-0195-y 29721701 PMC6779056

[16] Gerba CP, Betancourt WQ, Kitajima M. 2017. How much reduction of virus is needed for recycled water: a continuous changing need for assessment? Water Res 108:25–31. doi:10.1016/j.watres.2016.11.020 27838026 PMC7112101

[17] Chen Z, Ngo HH, Guo W. 2013. A critical review on the end uses of recycled water. Crit Rev Environ Sci Technol 43:1446–1516. doi:10.1080/10643389.2011.647788

[18] Association W. 2023. Water reuse in Florida. Available from: https://watereuse.org/wp-content/uploads/2023/06/Profiles-in-Reuse-Florida-1.pdf. Retrieved 15 Sep 2024.

[19] Fahrenfeld N, Ma Y, O’Brien M, Pruden A. 2013. Reclaimed water as a reservoir of antibiotic resistance genes: distribution system and irrigation implications. Front Microbiol 4:130. doi:10.3389/fmicb.2013.00130 23755046 PMC3664959

[20] Liguori K, Calarco J, Maldonado Rivera G, Kurowski A, Keenum I, Davis BC, Harwood VJ, Pruden A. 2023. Comparison of cefotaxime-resistant Escherichia coli and sul1 and intI1 by qPCR for monitoring of antibiotic resistance of wastewater, surface water, and recycled water. Antibiotics 12:1252. doi:10.3390/antibiotics12081252 37627672 PMC10451376

[21] Chern EC, Brenner K, Wymer L, Haugland RA. 2014. Influence of wastewater disinfection on densities of culturable fecal indicator bacteria and genetic markers. J Water Health 12:410–417. doi:10.2166/wh.2013.179 25252344

[22] Korajkic A, Kelleher J, Shanks OC, Herrmann MP, McMinn BR. 2022. Effectiveness of two wastewater disinfection strategies for the removal of fecal indicator bacteria, bacteriophage, and enteric viral pathogens concentrated using dead-end hollow fiber ultrafiltration (D-HFUF). Sci Total Environ 831:154861. doi:10.1016/j.scitotenv.2022.154861 35358531 PMC9291237

[23] Rosario K, Symonds EM, Sinigalliano C, Stewart J, Breitbart M. 2009. Pepper mild mottle virus as an indicator of fecal pollution. Appl Environ Microbiol 75:7261–7267. doi:10.1128/AEM.00410-09 19767474 PMC2786529

[24] Schmitz BW, Moriyama H, Haramoto E, Kitajima M, Sherchan S, Gerba CP, Pepper IL. 2018. Reduction of Cryptosporidium, Giardia, and fecal indicators by Bardenpho wastewater treatment. Environ Sci Technol 52:7015–7023. doi:10.1021/acs.est.7b05876 29847105

[25] Wu C, Zhou Y, Sun X, Fu L. 2018. The recent development of advanced wastewater treatment by ozone and biological aerated filter. Environ Sci Pollut Res Int 25:8315–8329. doi:10.1007/s11356-018-1393-8 29411279

[26] Hai FI, Riley T, Shawkat S, Magram SF, Yamamoto K. 2014. Removal of pathogens by membrane bioreactors: a review of the mechanisms, influencing factors and reduction in chemical disinfectant dosing. Water 6:3603–3630. doi:10.3390/w6123603

[27] Hiller CX, Hübner U, Fajnorova S, Schwartz T, Drewes JE. 2019. Antibiotic microbial resistance (AMR) removal efficiencies by conventional and advanced wastewater treatment processes: a review. Sci Total Environ 685:596–608. doi:10.1016/j.scitotenv.2019.05.315 31195321

[28] Lüddeke F, Heß S, Gallert C, Winter J, Güde H, Löffler H. 2015. Removal of total and antibiotic resistant bacteria in advanced wastewater treatment by ozonation in combination with different filtering techniques. Water Res 69:243–251. doi:10.1016/j.watres.2014.11.018 25497174

[29] Ottoson J, Hansen A, Björlenius B, Norder H, Stenström TA. 2006. Removal of viruses, parasitic protozoa and microbial indicators in conventional and membrane processes in a wastewater pilot plant. Water Res 40:1449–1457. doi:10.1016/j.watres.2006.01.039 16533517

[30] Eichmiller JJ, Borchert AJ, Sadowsky MJ, Hicks RE. 2014. Decay of genetic markers for fecal bacterial indicators and pathogens in sand from Lake Superior. Water Res 59:99–111. doi:10.1016/j.watres.2014.04.005 24793108

[31] Kim M, Wuertz S. 2015. Survival and persistence of host-associated Bacteroidales cells and DNA in comparison with Escherichia coli and Enterococcus in freshwater sediments as quantified by PMA-qPCR and qPCR. Water Res 87:182–192. doi:10.1016/j.watres.2015.09.014 26408951

[32] Varma M, Field R, Stinson M, Rukovets B, Wymer L, Haugland R. 2009. Quantitative real-time PCR analysis of total and propidium monoazide-resistant fecal indicator bacteria in wastewater. Water Res 43:4790–4801. doi:10.1016/j.watres.2009.05.031 19540546

[33] Elizaquível P, Aznar R, Sánchez G. 2014. Recent developments in the use of viability dyes and quantitative PCR in the food microbiology field. J Appl Microbiol 116:1–13. doi:10.1111/jam.12365 24119073

[34] Gomi R, Matsuda T, Matsui Y, Yoneda M. 2014. Fecal source tracking in water by next-generation sequencing technologies using host-specific Escherichia coli genetic markers. Environ Sci Technol 48:9616–9623. doi:10.1021/es501944c 25055157

[35] Senkbeil JK, Ahmed W, Conrad J, Harwood VJ. 2019. Use of Escherichia coli genes associated with human sewage to track fecal contamination source in subtropical waters. Sci Total Environ 686:1069–1075. doi:10.1016/j.scitotenv.2019.05.201 31200304

[36] USEPA. 2009. Method 1603: Escherichia coli (E. coli) in water by membrane filtration using modified membrane‐thermotolerant Escherichia coli agar (modifAied mTEC). US Environmental Protection Agency, Office of Water, Washington DC.

[37] Chern EC, Siefring S, Paar J, Doolittle M, Haugland RA. 2011. Comparison of quantitative PCR assays for Escherichia coli targeting ribosomal RNA and single copy genes. Lett Appl Microbiol 52:298–306. doi:10.1111/j.1472-765X.2010.03001.x 21204885

[38] Stachler E, Kelty C, Sivaganesan M, Li X, Bibby K, Shanks OC. 2017. Quantitative CrAssphage PCR assays for human fecal pollution measurement. Environ Sci Technol 51:9146–9154. doi:10.1021/acs.est.7b02703 28700235 PMC7350147

[39] Chern EC, Brenner KP, Wymer L, Haugland RA. 2009. Comparison of fecal indicator bacteria densities in marine recreational waters by QPCR. Water Expo Health 1:203–214. doi:10.1007/s12403-009-0019-2

[40] R Core Team. 2013. R: a language and environment for statistical computing.

[41] Helsel DR. 2011. Statistics for censored environmental data using Minitab and R. Vol. 77. John Wiley & Sons, Hoboken, NJ, USA.

[42] Lee L. 2017. CRAN-package NADA [WWW document]. Available from: https://cran.r-project.org/web/packages/NADA/index.html. Retrieved 13 Oct 2019.

[43] Agresti A. 2012. Categorical data analysis. Vol. 792. John Wiley & Sons.

[44] Oksanen J, Blanchet F, Friendly M, Kindt R, Legendre P, McGlinn D, Minchin P, O’hara R, Simpson G, Solymos P. 2016. vegan: community ecology package. R version 3.2. 4. Community ecol. packag. version 2.

[45] Kindt R. 2017. Package for community ecology and suitability. Analysis. R package version 2:8-3.

[46] Association W. 2023. Educate. Available from: https://watereuse.org/educate. Retrieved 15 Sep 2023.

[47] Eichmiller JJ, Hicks RE, Sadowsky MJ. 2013. Distribution of genetic markers of fecal pollution on a freshwater sandy shoreline in proximity to wastewater effluent. Environ Sci Technol 47:3395–3402. doi:10.1021/es305116c 23473470 PMC3629727

[48] Zimmer-Faust AG, Thulsiraj V, Lee CM, Whitener V, Rugh M, Mendoza-Espinosa L, Jay JA. 2018. Multi-tiered approach utilizing microbial source tracking and human associated-IMS/ATP for surveillance of human fecal contamination in Baja California, Mexico. Sci Total Environ 640–641:475–484. doi:10.1016/j.scitotenv.2018.05.172 PMC12045106 29864661

[49] Deng S, Yan X, Zhu Q, Liao C. 2019. The utilization of reclaimed water: possible risks arising from waterborne contaminants. Environ Pollut 254:113020. doi:10.1016/j.envpol.2019.113020 31421574

[50] Jäger T, Hembach N, Elpers C, Wieland A, Alexander J, Hiller C, Krauter G, Schwartz T. 2018. Reduction of antibiotic resistant bacteria during conventional and advanced wastewater treatment, and the disseminated loads released to the environment. Front Microbiol 9:2599. doi:10.3389/fmicb.2018.02599 30425704 PMC6218952

[51] Korajkic A, McMinn B, Herrmann MP, Sivaganesan M, Kelty CA, Clinton P, Nash MS, Shanks OC. 2020. Viral and bacterial fecal indicators in untreated wastewater across the contiguous United States exhibit geospatial trends. Appl Environ Microbiol 86:e02967-19. doi:10.1128/AEM.02967-19 32060019 PMC7117942

[52] Lou EG, Ali P, Lu K, Kalvapalle P, Stadler LB. 2023. Snapshot ARG removal rates across wastewater treatment plants are not representative due to diurnal variations. ACS EST Water 3:166–175. doi:10.1021/acsestwater.2c00467

[53] Kay D, Crowther J, Stapleton CM, Wyer MD, Fewtrell L, Edwards A, Francis CA, McDonald AT, Watkins J, Wilkinson J. 2008. Faecal indicator organism concentrations in sewage and treated effluents. Water Res 42:442–454. doi:10.1016/j.watres.2007.07.036 17709126

[54] Bunce JT, Robson A, Graham DW. 2020. Seasonal influences on the use of genetic markers as performance indicators for small wastewater treatment plants. Sci Total Environ 739:139928. doi:10.1016/j.scitotenv.2020.139928 32540662

[55] Morrison CM, Betancourt WQ, Quintanar DR, Lopez GU, Pepper IL, Gerba CP. 2020. Potential indicators of virus transport and removal during soil aquifer treatment of treated wastewater effluent. Water Res 177:115812. doi:10.1016/j.watres.2020.115812 32311575

[56] Li D, Van De Werfhorst LC, Steets B, Ervin J, Murray JLS, Blackwell A, Devarajan N, Holden PA. 2021. Sources of low level human fecal markers in recreational waters of two Santa Barbara, CA beaches: roles of WWTP outfalls and swimmers. Water Res 202:117378. doi:10.1016/j.watres.2021.117378 34246990

[57] Mayer RE, Bofill-Mas S, Egle L, Reischer GH, Schade M, Fernandez-Cassi X, Fuchs W, Mach RL, Lindner G, Kirschner A, Gaisbauer M, Piringer H, Blaschke AP, Girones R, Zessner M, Sommer R, Farnleitner AH. 2016. Occurrence of human-associated Bacteroidetes genetic source tracking markers in raw and treated wastewater of municipal and domestic origin and comparison to standard and alternative indicators of faecal pollution. Water Res 90:265–276. doi:10.1016/j.watres.2015.12.031 26745175 PMC4884448

[58] Xue J, Schmitz BW, Caton K, Zhang B, Zabaleta J, Garai J, Taylor CM, Romanchishina T, Gerba CP, Pepper IL, Sherchan SP. 2019. Assessing the spatial and temporal variability of bacterial communities in two Bardenpho wastewater treatment systems via Illumina MiSeq sequencing. Sci Total Environ 657:1543–1552. doi:10.1016/j.scitotenv.2018.12.141 30677920

[59] Wu Z, Greaves J, Arp L, Stone D, Bibby K. 2020. Comparative fate of CrAssphage with culturable and molecular fecal pollution indicators during activated sludge wastewater treatment. Environ Int 136:105452. doi:10.1016/j.envint.2019.105452 31931347

[60] McLellan SL, Huse SM, Mueller-Spitz SR, Andreishcheva EN, Sogin ML. 2010. Diversity and population structure of sewage‐derived microorganisms in wastewater treatment plant influent. Environ Microbiol 12:378–392. doi:10.1111/j.1462-2920.2009.02075.x 19840106 PMC2868101

[61] Gerba CP, Betancourt WQ, Kitajima M, Rock CM. 2018. Reducing uncertainty in estimating virus reduction by advanced water treatment processes. Water Res 133:282–288. doi:10.1016/j.watres.2018.01.044 29407709

[62] Blatchley ER, Gong W-L, Alleman JE, Rose JB, Huffman DE, Otaki M, Lisle JT. 2007. Effects of wastewater disinfection on waterborne bacteria and viruses. Water Environ Res 79:81–92. doi:10.2175/106143006x102024 17290975

[63] Simhon A, Pileggi V, Flemming CA, Bicudo JR, Lai G, Manoharan M. 2019. Enteric viruses in municipal wastewater effluent before and after disinfection with chlorine and ultraviolet light. J Water Health 17:670–682. doi:10.2166/wh.2019.111 31638019

[64] Stachler E, Akyon B, de Carvalho NA, Ference C, Bibby K. 2018. Correlation of crAssphage qPCR markers with culturable and molecular indicators of human fecal pollution in an impacted urban watershed. Environ Sci Technol 52:7505–7512. doi:10.1021/acs.est.8b00638 29874457

[65] Warish A, Triplett C, Gomi R, Gyawali P, Hodgers L, Toze S. 2015. Assessment of genetic markers for tracking the sources of human wastewater associated Escherichia coli in environmental waters. Environ Sci Technol 49:9341–9346. doi:10.1021/acs.est.5b02163 26151092

[66] Worley-Morse T, Mann M, Khunjar W, Olabode L, Gonzalez R. 2019. Evaluating the fate of bacterial indicators, viral indicators, and viruses in water resource recovery facilities. Water Environ Res 91:830–842. doi:10.1002/wer.1096 30848516 PMC6849880

[67] Elder RO, Keen JE, Siragusa GR, Barkocy-Gallagher GA, Koohmaraie M, Laegreid WW. 2000. Correlation of enterohemorrhagic Escherichia coli O157 prevalence in feces, hides, and carcasses of beef cattle during processing. Proc Natl Acad Sci U S A 97:2999–3003. doi:10.1073/pnas.97.7.2999 10725380 PMC16181

[68] Nopprapun P, Boontanon SK, Harada H, Surinkul N, Fujii S. 2020. Evaluation of a human-associated genetic marker for Escherichia coli (H8) for fecal source tracking in Thailand. Water Sci Technol 82:2929–2936. doi:10.2166/wst.2020.525 33341782

[69] Mayer RE, Reischer GH, Ixenmaier SK, Derx J, Blaschke AP, Ebdon JE, Linke R, Egle L, Ahmed W, Blanch AR, et al.. 2018. Global distribution of human-associated fecal genetic markers in reference samples from six continents. Environ Sci Technol 52:5076–5084. doi:10.1021/acs.est.7b04438 29570973 PMC5932593

[70] Caporaso JG, Lauber CL, Costello EK, Berg-Lyons D, Gonzalez A, Stombaugh J, Knights D, Gajer P, Ravel J, Fierer N, Gordon JI, Knight R. 2011. Moving pictures of the human microbiome. Genome Biol. 12:1–8. doi:10.1186/gb-2011-12-5-r50 PMC3271711 21624126

[71] Claesson MJ, O’Sullivan O, Wang Q, Nikkilä J, Marchesi JR, Smidt H, de Vos WM, Ross RP, O’Toole PW. 2009. Comparative analysis of pyrosequencing and a phylogenetic microarray for exploring microbial community structures in the human distal intestine. PLoS One 4:e6669. doi:10.1371/journal.pone.0006669 19693277 PMC2725325

[72] Eckburg PB, Bik EM, Bernstein CN, Purdom E, Dethlefsen L, Sargent M, Gill SR, Nelson KE, Relman DA. 2005. Diversity of the human intestinal microbial flora. Science 308:1635–1638. doi:10.1126/science.1110591 15831718 PMC1395357

