# Supplementary material for: Persistence of sewage-associated genetic markers in advanced and conventional treated recycled water: implications for microbial source tracking in surface waters
Source: mBio. 2024 Jun 12;15(7):e00655-24. doi: 10.1128/mbio.00655-24 (PMC11253620; doi:10.1128/mbio.00655-24)
Supplement: Supplemental material — Fig. S1 to S4; Tables S1 to S7. [file mbio.00655-24-s0001.pdf]

# Persistence of Sewage-Associated Genetic Markers in Advanced and Conventional Treated Recycled Water: Implications for Microbial Source Tracking in Surface Waters

Aldo E. Lobos[a], Javier F. Gallard-Góngora[b], Ruchi Korde[a], Eleanor Brodrick[a], Amanda M. Brandt[a], Valerie J. Harwood[a]

[a]Department of Integrative Biology, University of South Florida, Tampa, FL 33620, USA

[b]Department of Earth, Marine, and Environmental Sciences, Institute of Marine Science, University of North Carolina at Chapel Hill, Morehead City, NC 28557, USA

Table S1. Primer and probe concentrations and sequences, qPCR cycling parameters for assays used, and experiments where each assay was utilized.

| QPCR Targets | Primer/Probe sequences (Final concentration): 5'-3' | Cycling parameters | Reference | Experiment |
|---|---|---|---|---|
| H8 | forward primer (0.9 µM): ACAGTCAGCGAGATTCTTC<br>reverse primer (0.9 µM): GAACGTCAGCACCACCAA<br>probe (80 nM): FAM-ACTGGCATCGGCATGGAACAC-BHQ | 2 min at 50 °C, 10 min at 95 °C, followed by 40 cycles of (15 s at 95 °C, 60 s at 58 °C) | Senkbeil et al., 2019 | AWT and CWT persistence, and surface water survey |
| *uid*A | forward primer (1 µM): CAACGAACTGAACTGGCAGA<br>reverse primer (1 µM): CATTACGCTGCGATGGAT<br>probe (80 nM): VIC-CCCGCCGGGAATGGTGATTAC | 2 min at 50 °C, 10 min at 95 °C, followed by 40 cycles of (15 s at 95 °C, 60 s at 60 °C) | Chern et al., 2009 | AWT and CWT persistence, and surface water survey |
| EC23S857 | forward primer (1 µM): GGTAGAGCACTGTTTTGGCA<br>reverse primer (1 µM): TGTCTCCCGTGATAACTTTCTC<br>probe (80 nM): FAM-TCATCCCGACTTACCAACCCG-TAMRA | 10 min at 95 °C followed by 40 cycles of (15 s at 95 °C and 60 s at 56 °C) | Chern et al., 2011 | AWT and CWT persistence |
| HF183/ BacR287 | forward primer (1 µM): ATCATGAGTTCACATGTCCG<br>reverse primer (1 µM): CTTCCTCTCAGAACCCCTATCC<br>Bac234IAC probe (80 nM): VIC-AACACGCCGTTGCTACA-MGB<br>BacP234MGB (80 nM): [6-FAM]-CTAATGGAACGCATCCC-MGB | 2 min at 50 °C, 10 min at 95 °C followed by 40 cycles of (15 s at 95 °C and 60 s at 60 °C) | USEPA, 2019 | Discharge study, AWT and CWT persistence, and surface water survey |
| CrAssphage CPQ-056 | forward primer (1 µM): CAGAAGTACAAACTCCTAAAAAACGTAGAG<br>reverse primer (1 µM): GATGACCAATAAACAAGCCATTAGC<br>probe (80 nM): FAM-AATAACGATTTACGTGATGTAAC-MGB | 10 min at 95°C followed by 40 cycles of (15 s at 95 °C and 1 min at 60 °C) | Stachler et al., 2017 | AWT and CWT persistence |

Table S2. QPCR assay amplicon length and sequences utilized in gBlocks™ material for standard curves.

| Assay | Amplicon length of qPCR target (bp) | Gene sequence (5'-3') |
|---|---|---|
| H8 | 177 | ACAGTCAGCGAGATTCTTCGCCACGCCGGCGTGGCG CATCTGCTGCTGGAGGCGGACGCGCAGAAGGTCGAG GCCGCGCGTGCCGCCGGCGCGCCGGTGTTCCATGCC GATGCCAGTCGGCCCGATACCTTGCTGGCTGCCGGC TTGACGCATGCACACTTGGTGGTGCTGACGTTC |
| EC23S857 | 88 | GGTAGAGCACTGTTTTGGCAAGGGGGTCATCCCGAC TTACCAACCCGACTCGAGCTGCGAATACCGGAGAAA GTTATCACGGGAGACA |
| HF183/ BacR287 | 132 | ATCATGAGTTCACATGTCCGCATGATTAAAGGTATTT TCCGGTAGACGATGGGGATGCGTTCCATTAGCTCGA GATAGTAGGCGGGGTAACGGCCCACCTAGTCAACGA TGGATAGGGGTTCTGAGAGGAAG |
| CrAssphage CPQ-056 | 126 | CAGAAGTACAAACTCCTAAAAAACGTAGAGGTAGA GGTATTAATAACGATTTACGTGATGTAACTCGTAAA AAGTTTGATGAACGTACTGATTGCAACAAAGCTAAT GGCTTGTTTATTGGTCATC |

Table S3. Statistical comparisons of microbial variables measured by qPCR in untreated sewage from AWT and CWT facilities. Data are expressed as concentration ($log_{10}$ GC/100 mL). Data from like facilities (AWT or CWT) were pooled (n=9). Variables were individually compared between AWT and CWT facilities by Dunn rank sum tests with Bonferroni correction. Differences in frequency of detection for culturable EcH8 were compared by Fisher's Exact test. P-values < 0.05 are bolded.

| Microbial Variables | P value: AWT vs CWT |
|---|---|
| Culturable *E. coli* | 0.2670 |
| Culturable EcH8 | 0.1033 |
| EC23S857 | **0.0013** |
| HF183 | 0.1440 |
| H8 | 0.0849 |
| CPQ_056 | **0.0017** |

Table S4. $Log_{10}$ reduction values (mean ± standard error) of all qPCR targets in conventional (CWT) and advanced (AWT) wastewater treatment facilities (n=3).

| **Conventional** | **EC23S857** | **HF183** | **H8** | **CPQ_056** |
|---|---|---|---|---|
| A | 4.67 ± 0.64 | 4.01 ± 0.99 | 4.35 ± 0.96 | 1.72 ±0.31 |
| B | 4.70 ± 1.25 | 4.55 ± 1.31 | 4.32 ± 1.09 | 1.93 ± 0.07 |
| C | 5.77 ± 0.03 | 5.58 ± 0.18 | 5.57 ± 0.25 | 1.82 ± 0.20 |
| **Advanced** | | | | |
| D | 4.75 ± 0.57 | 4.12 ±0.13 | 3.74 ± 0.36 | 5.17 ± 0.35 |
| E | 6.61 ± 0.39 | 6.42 ± 0.63 | 6.08 ± 0.16 | 6.82 ± 0.12 |
| F | 5.00 ± 1.28 | 6.85 ± 0.16 | 5.91 ± 0.05 | 4.50 ± 1.20 |

Table S5. *P* values for statistical comparisons among microbial variables measured by qPCR in pooled untreated sewage and recycled water data (n=18). P-values < 0.05 are bolded.

| **qPCR Variables** | **Untreated Sewage** | **Recycled Water** | | |
|---|---|---|---|---|
| | **Median $log_{10}$ concentration** | **Median log10 concentration** | **Frequency of detection** | **$Log_{10}$ reduction** |
| EC23S857 : HF183 | 0.0512 | 0.3390 | **0.0455** | > 0.9999 |
| EC23S857 : H8 marker | **< 0.0001** | 0.0765 | **0.0191** | > 0.9999 |
| EC23S857 : CPQ_056 | **0.0021** | 0.3170 | 0.1040 | 0.0832 |
| H8 marker : HF183 | **< 0.0001** | 0.2610 | 1.0000 | > 0.9999 |
| H8 marker : CPQ_056 | **0.0017** | **0.0084** | 0.7110 | 0.3740 |
| HF183 : CPQ_056 | 0.2430 | 0.0744 | 1.0000 | 0.1280 |

Table S6. Significant relationships among concentrations of microbial variables in untreated sewage and recycled water pooled data (n=18). Kendall's tau reflects the ordinal association of the data; higher values indicate greater correlation. P-values < 0.05 are bolded.

| **qPCR Variable 1** | **qPCR Variable 2** | **Untreated Sewage** | | **Recycled Water** | |
|---|---|---|---|---|---|
| | | p-value | tau | p-value | tau |
| EC23S857 | HF183 | 0.1751 | 0.24 | **0.0005** | 0.62 |
| EC23S857 | H8 marker | **0.0006** | 0.57 | **0.0136** | 0.44 |
| EC23S857 | CPQ_056 | **0.0067** | 0.46 | 0.2690 | 0.19 |
| H8 marker | HF183 | **0.0022** | 0.52 | **0.0001** | 0.74 |
| H8 marker | CPQ_056 | **0.0085** | 0.45 | 0.1062 | 0.29 |
| HF183 | CPQ_056 | 0.0573 | 0.33 | 0.0760 | 0.32 |

Table S7. Surface water survey and the frequency of detection for HF183 and culturable EcH8 for each site. All samples were collected monthly between 8/11/2020 and 8/9/2022.

| Surface Water Sites | Water Type | Number of samples tested | HF183 frequency of detection (%) | EcH8 frequency of detection (%) |
|---|---|---|---|---|
| S1 | Freshwater | 16 | 68.75 | 6.25 |
| S2 | Estuarine | 17 | 100 | 11.76 |
| S3 | Estuarine | 16 | 93.75 | 6.25 |
| S4 | Estuarine | 8 | 100 | 0.00 |
| S5 | Freshwater | 4 | 0.00 | 0.00 |
| S6 | Marine | 16 | 81.25 | 12.50 |
| S7 | Freshwater | 14 | 100 | 64.29 |
| S8 | Marine | 12 | 58.33 | 16.67 |
| Total | | 103 | 82.50 | 16.50 |

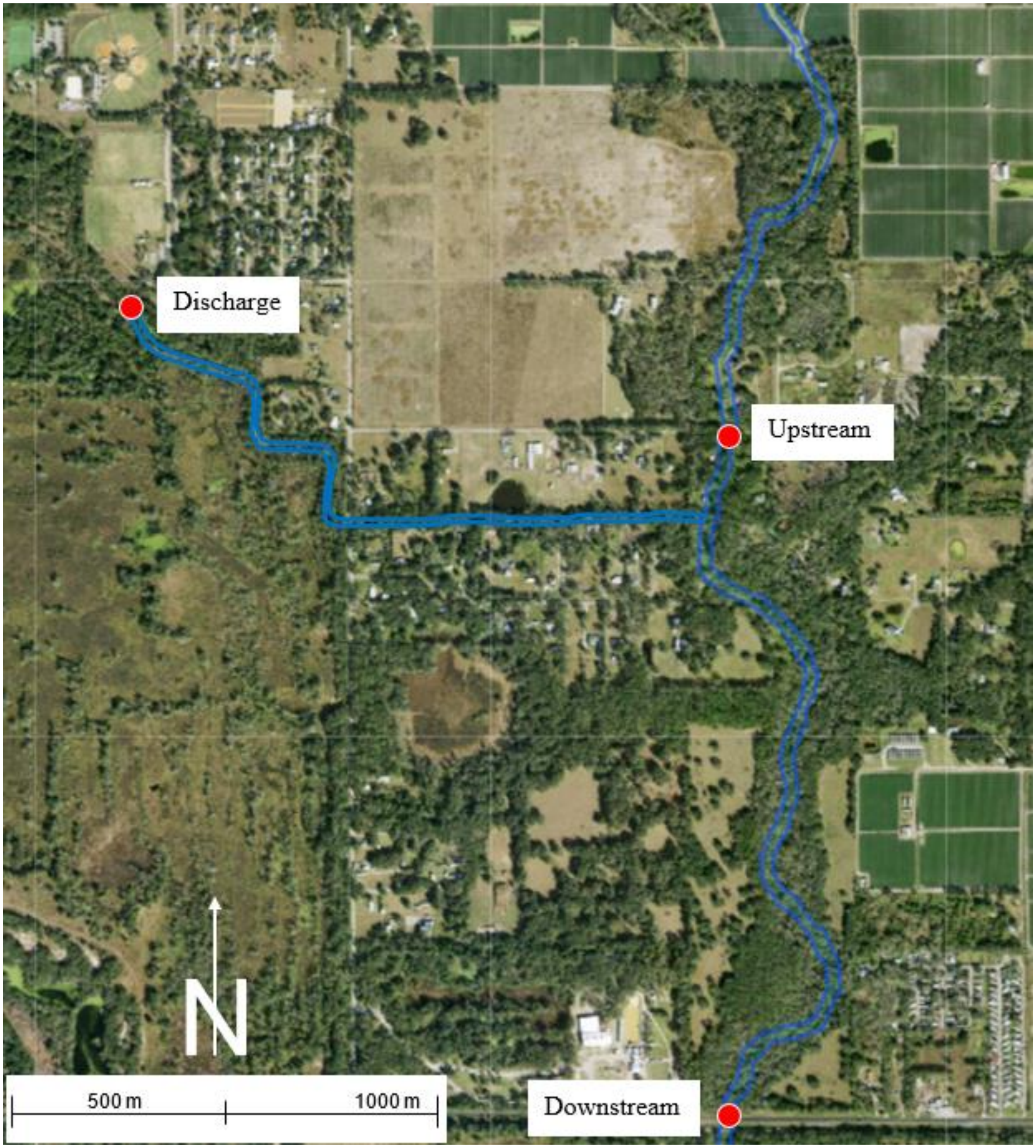


Figure S1. Map showing the sampling locations of the field study. Recycled water travels from the discharge site along a canal to enter Turkey Creek. The length that the treated effluent travels from the discharge site to the downstream site is 3.22 km. The upstream site is 0.24 km upstream of the confluence and is not affected by the discharge. Tampa Bay Water Atlas (https://tampabay.wateratlas.usf.edu/waterbodies/rivers/74/).

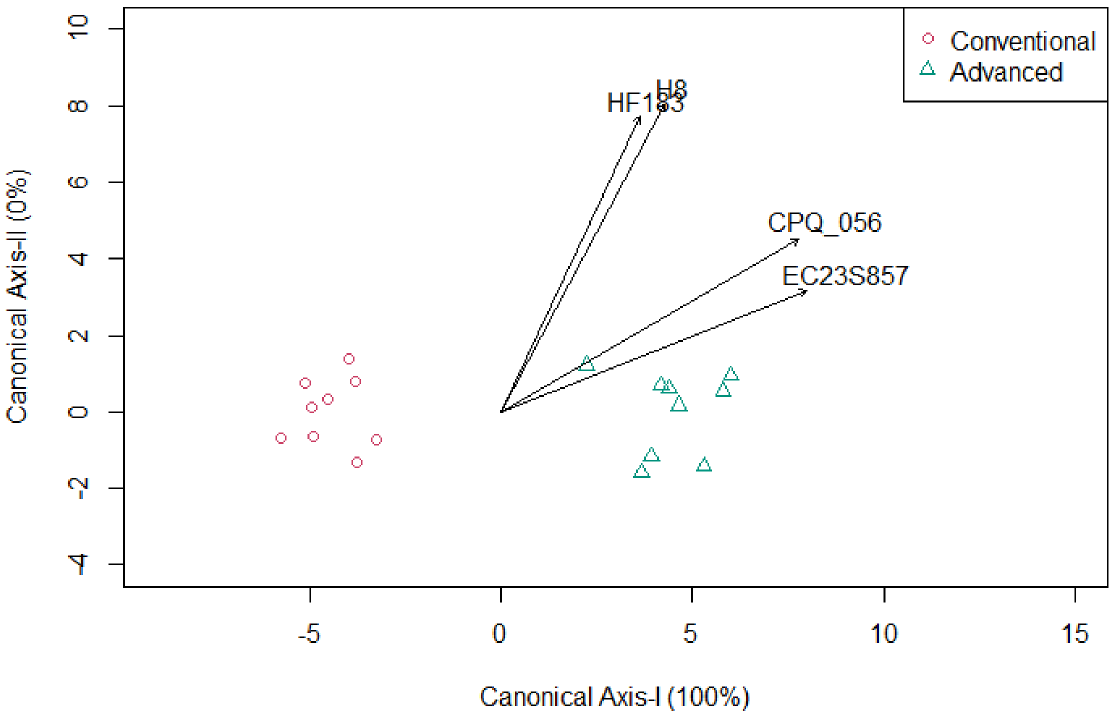


Figure S2. Relationships among microbial variables measured by qPCR in untreated sewage collected from AWT and CWT facilities (conventional = red circles, and advanced = green triangles) analyzed by canonical analysis of principal coordinates and linear discriminant analysis. Canonical axis I (horizontal) explained 100% of the variability, while canonical axis II (vertical) explained 0% of the variability observed. Microbial variables were significantly greater in untreated sewage from AWT compared to CWT facilities (p = 0.002).

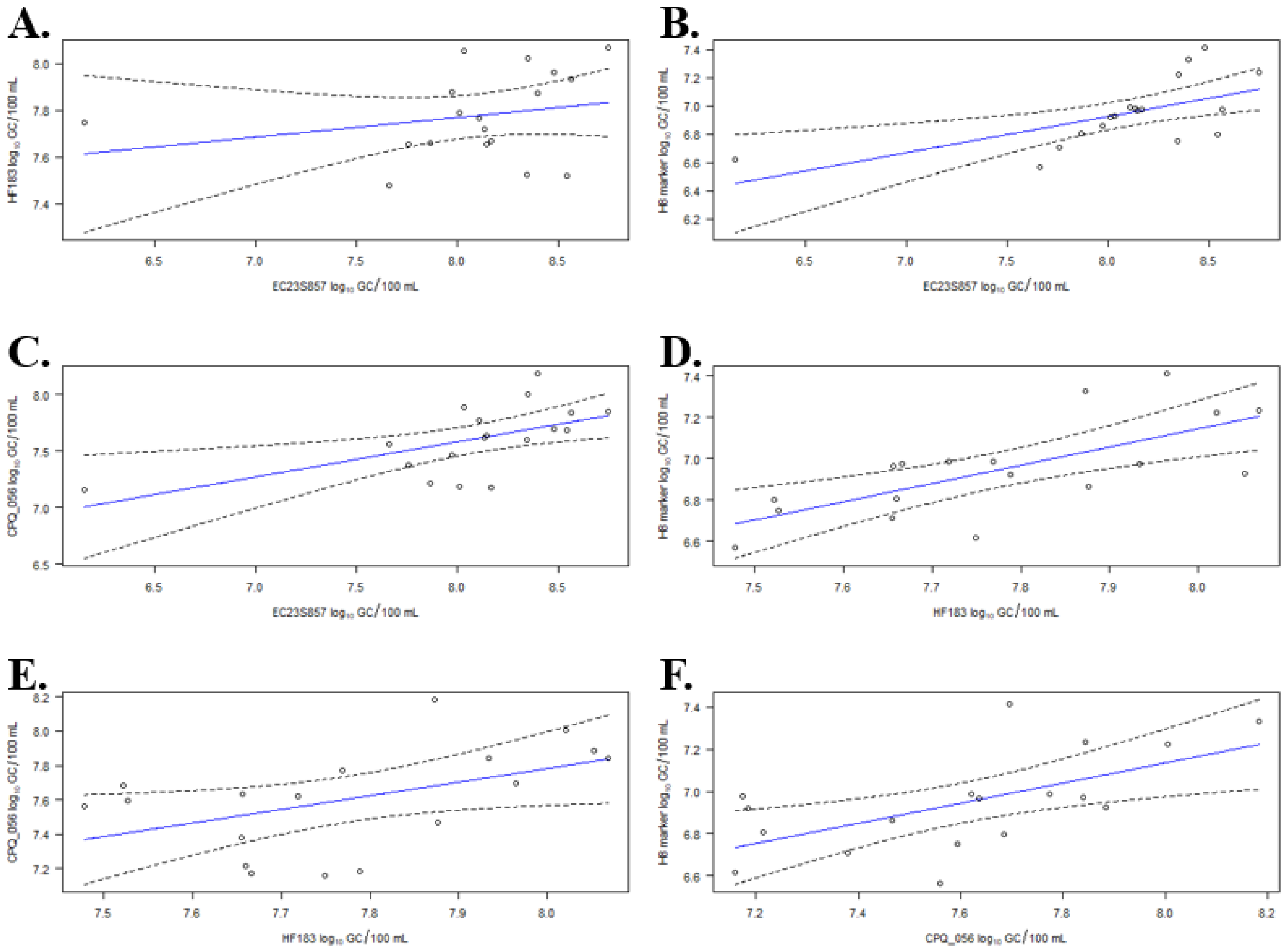


Figure S3. Relationships among microbial variables in pooled (AWT and CWT) untreated sewage data. The solid line depicts the simple linear regression relationship (95% confidence interval shown by dashed lines). HF183 and EC23S857 (A), H8 and EC23S857 (B), CPQ_056 and EC23S857 (C), H8 and HF183 (D), CPQ_056 and HF183 (E), H8 and CPQ_056 (F).

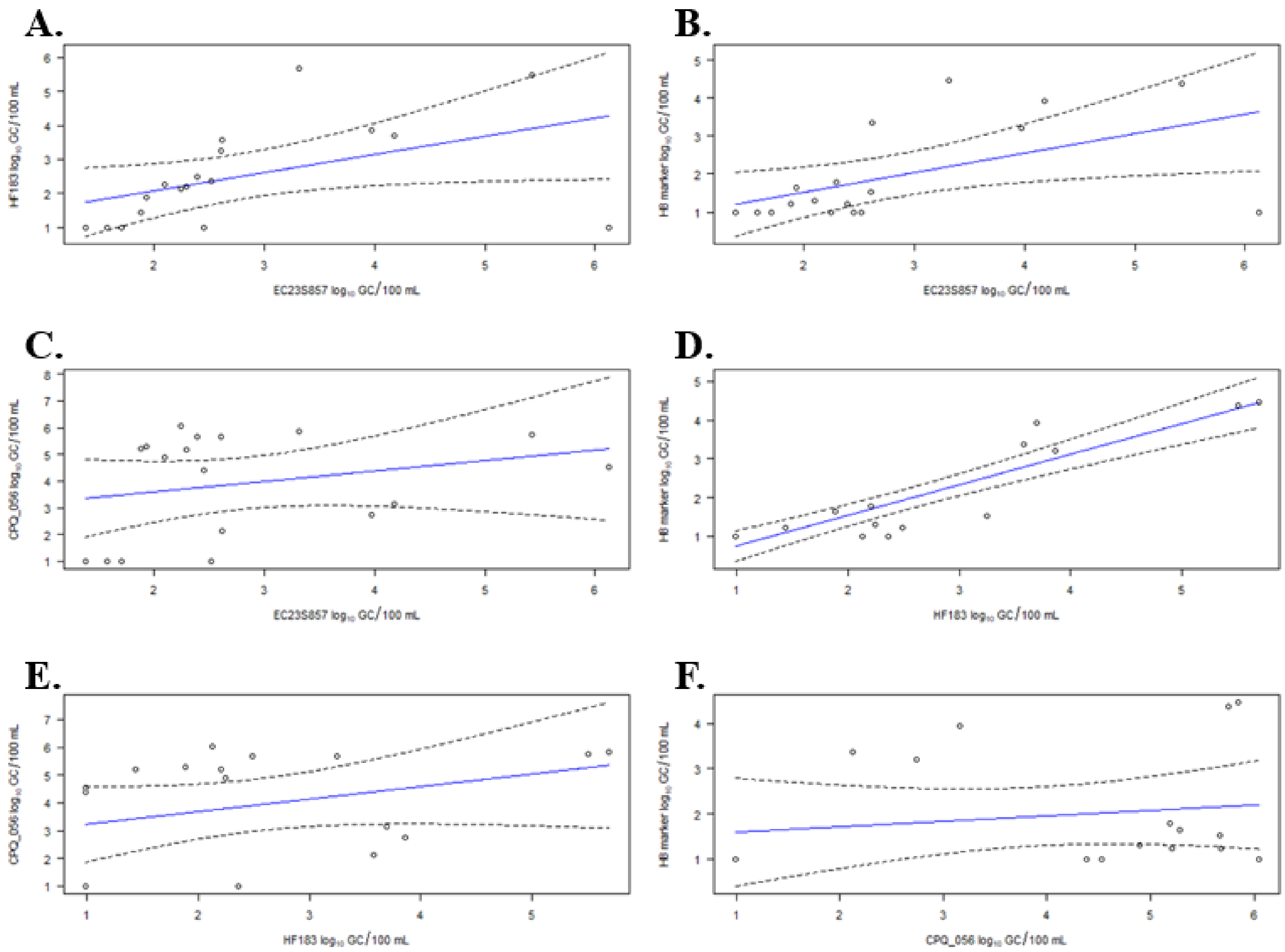


Figure S4. Relationships among microbial variables in pooled (AWT and CWT) recycled water data. The solid line depicts the simple linear regression relationship (95% confidence interval shown by dashed lines). HF183 and EC23S857 (A), H8 and EC23S857 (B), CPQ_056 and EC23S857 (C), H8 and HF183 (D), CPQ_056 and HF183 (E), H8 and CPQ_056 (F).
